# ‘The fish that stop’: drivers of historical decline for Pacific cod and implications for modern management in an era of rapidly changing climate

**DOI:** 10.1098/rstb.2024.0278

**Published:** 2025-07-10

**Authors:** Loren McClenachan, Bruce Anderson, Jason Addison, Steve Barbeaux, Karoline Moore, Kai Muir, Katherine Reedy, Ingrid Spies, Catherine F. West

**Affiliations:** ^1^ Department of History, University of Victoria, Victoria, British Columbia, Canada; ^2^ School of Environmental Studies, University of Victoria, Victoria, British Columbia, Canada; ^3^ Department of Earth and Environment, Boston University, Boston, MA, USA; ^4^ US Geological Survey, Moffet Field, CA, USA; ^5^ Alaska Fishery Science Center, Seattle, WA, USA; ^6^ Department of Anthropology, Idaho State University, Pocatello, ID, USA; ^7^ Department of Anthropology and Archaeology Program, Boston University College of Arts and Sciences, Boston, MA, USA

**Keywords:** historical ecology, Pacific cod, climate change, overfishing, marine heatwaves, climate history

## Abstract

In the Gulf of Alaska, a series of marine heat waves depleted Pacific cod (*Gadus macrocephalus*) biomass to the lowest abundance ever recorded and led to the fishery’s closure in 2020. Although the fishery has been productive for decades, this collapse may have historical precedents. Traditional knowledge holders refer to cod as ‘the fish that stop’, and there is a suggested period of decline in the 1930s. Here we conduct a catch reconstruction of the early commercial fishery (1864–1950), confirming a rapid catch decline in the 1920s and 1930s. Next, we evaluate evidence for possible drivers. We document changes to demand and technology that contributed to declining catch. However, we also find both qualitative and quantitative evidence of depletion, suggesting catch declines were not driven entirely by social factors. Overfishing may have contributed to localized catch declines as evidenced by declining catch rates in heavily fished localities. We also find evidence for climate as a driver of regional decline, with the period of catch decline characterized by up to 2°C higher temperatures as compared to the earlier period of high fisheries production. Our analysis underscores the importance of understanding long-term drivers of fisheries productivity and the value of linking fisheries and climate histories.

This article is part of the theme issue ‘Shifting seas: understanding deep-time human impacts on marine ecosystems’.

## 1. Introduction

A growing body of research in historical ecology has demonstrated past changes in marine fisheries [1]. To date, historical fisheries research has focused most on past fishing and its impacts on fish populations and marine ecosystems [2–5]. Although these insights have proved invaluable and relevant to management [6–8], some of the largest management challenges today relate to the combined effects of fishing and climate change, with an increasingly pressing need to understand the impacts of warming waters on marine ecosystems and fisheries over long time scales [9]. Therefore, employing methods of historical ecology towards understanding the complex drivers of change to past fisheries, including climate forcing, may inform the response of fish stocks to the current climate crisis.

Warming waters and marine heatwaves are increasing worldwide [10], with effects on marine fisheries including changes to migration patterns, distribution, phenology and metabolic rate of targeted species [11]. For example, the movement of marine animals poleward or to greater depths following optimal temperature conditions is a well-documented effect of warming waters [12,13]. Marine heatwaves have particularly pronounced impacts, including disruptions to reproduction, recruitment and survival, all of which affect the overall abundance of marine species and the productivity of associated fisheries. While the human-induced climate impacts on ocean temperatures are arguably limited to the past half century, periods of rapid temperature change occurred prior to that time, with impacts on marine fisheries and the coastal communities that have depended on them [9,10]. In particular, warming in the early twentieth century has been linked to well-known phenomena such as the Dust Bowl [14]. Such past periods of relatively rapid temperature change can provide an analogue to modern warming and a window into how complex ecological drivers affected marine fisheries over long time scales.

The Pacific cod fishery in Alaska provides an ideal case study to understand the effect of multiple drivers of decline over long time scales, including climate forcing. The fishery experienced recent climate-driven declines, with marine heatwaves in 2015 and 2016 resulting in extreme declines in abundance prompting fisheries closures [15]. Cod life history is sensitive to warming at several stages, with reproduction and egg survival affected by temperatures at depth and offshore in the winter and early spring, and shallow, inshore water (0−20 m) temperatures in the spring, summer and autumn impacting larvae, juveniles and adults [16–19]. Marine heatwaves can trigger early reproduction, which is linked to reduced survival [17]. Cod biomass declines during the recent marine heatwaves have been attributed to an increase in metabolic demand and reduced prey supply [15].

While the Pacific cod fishery is currently the second largest fishery in Alaska and is considered to be well-managed [15,20], this recent collapse may have precedents in history. The Aleut name for Pacific cod, *atxida* or *atxidan*, translates to ‘the fish that stops’ and communicates the spatial inconsistency of the fishery [21]. The 1930s has been indicated as a period of possible decline in the modern scientific literature [16], but the existence, magnitude and drivers of this decline are poorly understood.

Surprisingly little has been written about the commercial Pacific cod fishery in the late nineteenth and early twentieth century, probably because it operated in the shadow of both the globally dominant Atlantic cod (*Gadus morhua*) fishery and the regionally dominant Pacific salmon (*Oncorhynchus* spp.) fishery [5,22]. In comparison, the Pacific cod fishery was a relatively smaller industry whose demand fluctuated in response to the availability of Atlantic cod [23,24]. Here we conduct a catch reconstruction of the commercial fishery for Pacific cod (1864−1950) in order to assess the existence and magnitude of the reported decline in the 1930s. We then evaluate three possible drivers of declining catches in this period: social change, overfishing and climate change. This work aims to address the question of how diverse and complex drivers of change interact to affect fisheries dynamics over century-long time scales.

### (a) A brief history of the Pacific cod fishery

**Figure 1.**
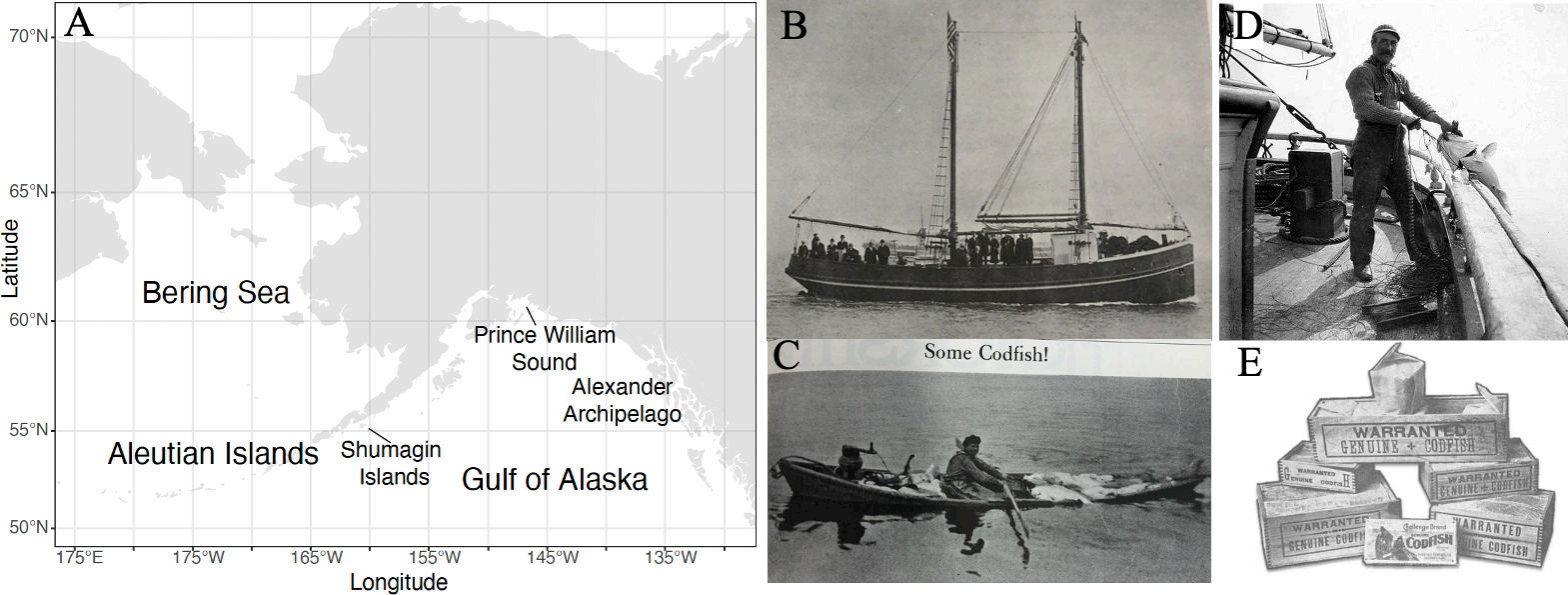
Images from the American salt cod fishery. (A) Map of the region with place names mentioned in the text. (B) Alaska Codfish Company’s schooner ‘Alasco’ on her trial trip [25]. (C) A dory fisherman returning with a load of fish, in a news article titled ‘Some Codfish!’ [26]. (D) An American fisherman in Alaska, *ca* 1910−1930, University of Washington Archives, John Cobb Collection. (E) Advertisement for salt cod sold by the Robinson Fisheries Company, Anacortes, Washington [27]. Photograph used with permission from the University of Washington Archives; map created using R Statistical Software and Adobe Illustrator.

The first commercial export fishery for Pacific cod began in 1864 in the North Pacific (figure 1A). The early fishing fleet included sail-powered wooden vessels, with schooner rigs being most common (figure 1B). These larger vessels carried small wooden dories that were designed to be nested inside each other for transport on deck. Fishermen targeted cod from these rowed dories using handlines (figure 1C,D). Beginning in the 1910s, both schooners and dories began to be equipped with gasoline motors. Fish were dried and sold as salt cod (figure 1E).

A vessel-based fishery operated in the Sea of Okhotsk, the Bering Sea (BS), the Gulf of Alaska (GOA) and the Aleutian Islands (AI). One key fishing region was the Shumagin Islands, located in the current-day GOA management area (electronic supplementary material, figure S1). A shore-station fishery included more than 80 stations located across the AI, Prince William Sound and Alexander Archipelago (figure 1A). The vessel fishery operated primarily in the summer months, when cod returned to the offshore banks to feed after spawning during winter months, while shore stations (using the same dories deployed by vessels) targeted cod that were abundant on the nearshore banks year-round [23]. Minimum length requirements set by the salt cod fish companies ranged from 23 inches (58.42 cm) to 28 inches (71.12 cm), with a 28-inch minimum set by one of the largest companies [28].

This schooner fishery in the BS and GOA was dominated by American vessels based in San Francisco and Puget Sound (ranging from 1 to 23 vessels annually), with two British Columbia-based vessels active in select years (1902−1905 and 1913−1914). Japanese vessels also engaged in schooner fishing, selling fish to American companies throughout the 1910s and 1920s. Japanese fishing focused on the Sea of Okhotsk, but between 1917 and 1923, Japanese schooners were observed fishing in the AI and the BS [29]. American schooners transported fish from both the vessel- and shore-station fisheries to ports in San Francisco and Puget Sound.

In the 1930s, the fishery began to transition to a trawling fleet. Several American trawling experiments before the 1940s were reported to be unsuccessful owing to the rocky terrain of the sea floor [30]. The Japanese trawling fishery developed in the 1930s, with fishing in the BS intensifying after 1933 [31]. The American trawling fishery began experimentally in 1940 and commenced in earnest in 1946, though was very limited [23]. Cod fisheries remained limited in the 1950s–1970s, and cod were targeted as bait for crab and halibut. In the late 1970s, an increase in targeted cod fishing followed the ‘Americanization’ of fisheries in Alaska. Crashes in Atlantic cod populations in the 1990s led to increased demand, and Pacific cod landings grew to an average of more than 250,000 ton annually in the twenty-first century [23,32].

## 2. Methods

### (a) Catch reconstruction

Catch reconstructions are used to characterize past fisheries in the absence of robust landing data. This method relies on a diversity of data types, ranging from reported catch data to estimates of per-capita consumption [33]. Catch reconstructions have been used within the stock assessment process in order to account for the full time series of removals and have also been used to estimate catch in data-limited regions with the goal of improving global and national catch statistics, typically after 1950 [34,35]. They have also been used in historical ecology analyses over longer time frames; for example, estimating fish catch over centuries [36,37].

The goal of our catch reconstruction was to identify the magnitude of the fishery and evaluate the existence, timing and magnitude of the possible period of decline in the early twentieth century. Therefore, we focused on the period from the start of the commercial export fishery in 1864 through to the end of the salt cod fishery in 1950. Our catch reconstruction focuses specifically on three regions and areas currently managed by the United States: the BS, AI and GOA (electronic supplementary material, figure S1). Owing to inconsistent data availability, we exclude the Sea of Okhotsk, which has been managed by Russia since 1892 [23].

A summary of the fisheries included in the reconstruction, as well as a list of all data sources used, is found in the electronic supplementary material, tables S1 and S2, alongside a more detailed description of our catch reconstruction methods. The majority of the catch derives from the American schooner fishery, for which catch data were available consistently. Several sources document catch, but only one [24] distinguished the Sea of Okhotsk catch from our study region (BS, AI and GOA) before 1910; therefore, we used these catch values from 1864 to 1909. We estimated Japanese schooner catch based on observed catch rates, effort and estimates of catch in our region of interest (electronic supplementary material, table S2). We estimated Japanese trawl catch from observations of catch rate and effort (1930−1932; electronic supplementary material, table S2) and foreign groundfish catch reporting (1933−1950) [32]. Catch data were available for the American trawl fishery beginning in 1940 (electronic supplementary material, table S2).

Early catch was reported in numbers of fish, which we converted to live weight using a conversion ratio of 11 pounds (4.99 kg) per fish, based on the estimated weight of fish ranging from 28 to 30 inches (71.12–76.2 cm) in length (electronic supplementary material, table S3). We converted salted weight to live weight for one year (1943), using a ratio of 1 : 3.5, which is the average value from two sources, one from the salt-cod era [38] and one based on 1980s industry data [23].

We conducted time-series analyses of our catch reconstruction using the palaeontological statistics software package PAST (v.4.17) [39]. Analytical approaches included: (i) a multi-taper method (MTM) for spectral analysis [40], which identifies the dominant frequencies of the entire dataset and allows for significance testing; and (ii) a continuous wavelet transform (CWT) to examine changes in the spectral power as a function of time [41]. The MTM is a spectral density estimation approach that distinguishes between stochastic noise in climatic datasets and statistically significant signals with a periodicity by applying a set of tapering window functions that produce a spectrum with low spectral leakage, low variance and minimizes end effects at the beginning and end of the dataset. The CWT expands on this approach by allowing for spectral analysis that both identifies dominant modes of variability while at the same time allowing for examining how these modes vary in time, as well as permits significance testing by comparing against a univariate lag-1 autoregressive (red-noise) process.

### (b) Observations of depletion and drivers of decline

We assessed the evidence for three possible drivers of decline: social changes, overfishing and climate. Our analysis of the social drivers of decline used first-hand accounts of the fishery, the most relevant of which were articles published in the *Pacific Fisherman* magazine. Published by and for the fishing industry, these articles describe contemporary issues such as fishing activity, changes in technology, labour relations and market development. We reviewed all issues of *Pacific Fisherman* from 1915 to 1940. All articles related to Pacific cod were collected, transcribed and categorized as providing information on observations of depletion in the water or possible drivers of change in the fishery.

We assessed qualitative and quantitative evidence that reductions in catch were driven by depletion in the water in several ways. First, qualitative descriptions of decline were extracted from the *Pacific Fisherman* and other contemporary sources. These included observations of poor fishing seasons or descriptions of declines in particular locations as compared to previous seasons. We also extracted information on fish size and reports of fish size being larger or smaller than previous years, as a metric of fisheries health. For observations of fish size, we recorded the number of articles in each year noting a size increase or decrease, as well as any relevant descriptions of these changes.

Second, we conducted quantitative estimates of catch per unit effort (CPUE) across the American salt cod fishery, using vessel tonnage and annual fish catch from 1884 to 1925, which was provided in a 1927 US government report [24]. Vessel size directly influenced the number of fishing dories that a vessel could carry and therefore is considered a good metric of fishing power. We excluded gas vessels (1915–1925), owing to their increased fishing power. Although this analysis is limited by data availability on vessel tonnage ending in 1925, information on fishing grounds targeted allowed an analysis of catch per vessel tonne across subregions. For this analysis, we separated the Shumagin Islands from all other regions, which were later combined in a region Cobb called the Alaska Banks [24]. Finally, to assess the likelihood of overfishing as a driver of decline in the early twentieth century, we compared catch totals from our catch reconstruction to annual catches in the modern fishery.

We assessed climate drivers of decline by evaluating spatial and temporal trends in water temperature. For this analysis, we use data from the Simple Ocean Data Assimilation—sparse input v. 3 (SODAsi.3). These data span the period 1815−2013 and consist of monthly mean values on a 0.4° × 0.25° longitude/latitude grid and on 40 vertical levels. For our spatial analysis, we considered both sea surface temperatures (SSTs) and temperatures across the water column to 300 m in depth (T300). We further disaggregated surface temperature and water column temperature anomalies across three seasons, spring (February–April), summer (May–July) and autumn (August–October). These seasons were selected to represent cod life history, with spawning in the spring, before migration in the summer and autumn, with a key life-history component being survival of the first winter, which is influenced by autumn temperatures [19]. We evaluated spatial patterns in temperature by comparing temperatures during the period prior to and during high fishery productivity (1895−1915) with the subsequent period of low productivity (1925−1945). For this analysis, we considered time periods 5 years prior to the periods of maximum productivity and decline with the assumption that any temperature effects would have the largest impacts on reproduction and recruitment, with a 5-year lag before cohorts of fish matured into the fishery.

For our analysis of temporal trends, we focused on SST anomalies along the continental shelf south of the Alaska Peninsula centred on the Shumagin Islands in the western GOA where the fishery was known to be active. We considered SST anomalies for the timespan that corresponded to our catch reconstruction (1864−1950), as well as for the entirety of available data (1851−2013).

## 3. Results

### (a) Catch reconstruction

**Figure 2.**
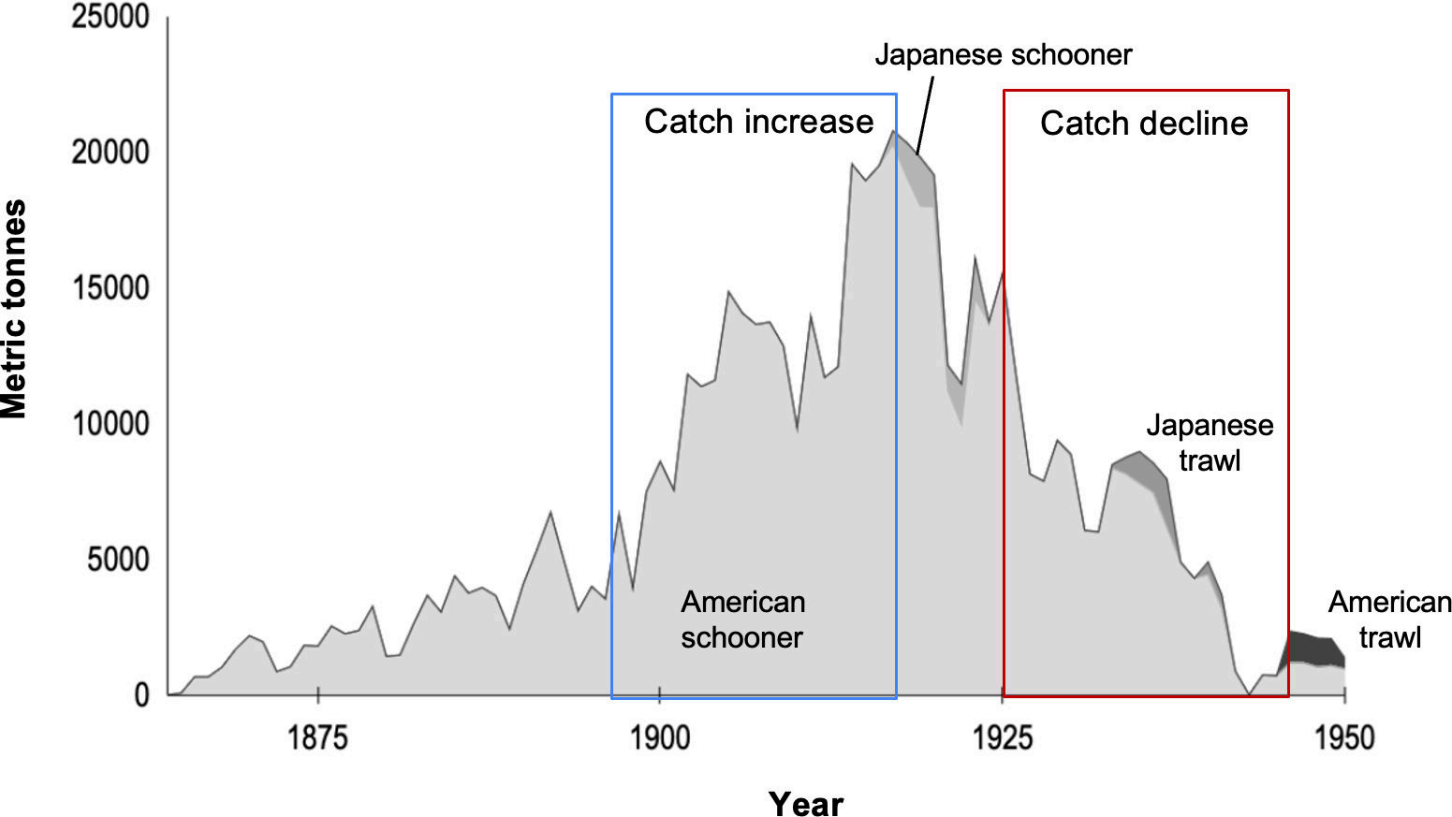
Pacific cod catch reconstruction for the BS, AI and GOA, 1864−1950. Periods of catch increase (1895−1915) and catch decline (1925−1945) correspond to the periods used for analysis in figure 5, with a 5-year time lag to account for the time it would take for affected year classes to mature into the fishery.

Commercial cod catch from our three regions was variable but increased from 1864 to 1919 (figure 2). Annual catch first exceeded 10 000 metric tonnes (t) in 1902 and reached a peak of 20 293 t in 1919. The majority of this catch came from the American schooner fishery, with the Japanese schooner, Japanese trawl and American trawl fisheries contributing a relatively small amount throughout this time period. Catches were variable but declined from 1920 to 1943. Annual catches dropped below 10 000 t in 1927, reaching a low of 18.9 t in 1943. The largest annual decline occurred in 1921 with a 33% decline from the previous year. Together, 1926 and 1927 represented the largest sustained reduction, declining 19 and 18%, respectively, with catches dropping to less than half the peak values and not recovering. The schooner fishery is responsible for the steady increase in removals during the period of increase, followed by subsequent catch declines, even though the fishery was probably becoming more efficient as mechanized vessels came online.

The American schooner fishery included both vessel-based catch and catch from fixed shore stations. Shore-station catch represented a substantial percentage of total catches, particularly in the first five decades of the fishery. Shore-station catch was equivalent to vessel catches until 1908, when vessel catches grew quickly, remaining above shore-station catches until 1919 (electronic supplementary material, figure S2). The peak catch in shore stations occurred in 1920 before declining throughout that decade.

### (b) Time-series analysis

Both of our time-series analyses demonstrate significant cycles in the catch data. The first is a multi-decadal cycle (22–40 years), and the second is a shorter decadal cycle (10–15 years). This second decadal cycle can be seen in the smaller increases and decreases overlain on the overall trend, increasing in amplitude until 1923 and then declining to the end of the dataset in 1950 (see the electronic supplementary material, figure S3 for more details).

### (c) Social drivers of decline

**Figure 3.**
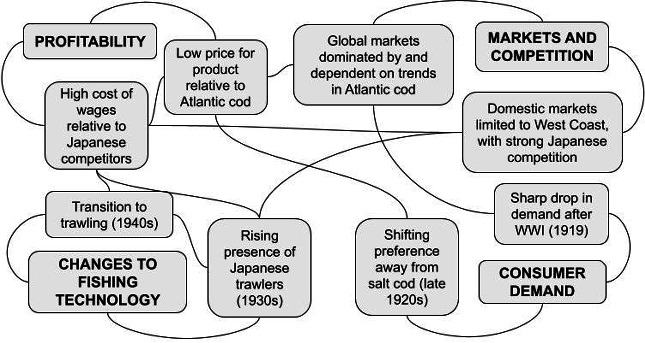
Social factors that contributed to decline of the Pacific cod fishery, 1915−1940.

Several inter-related social factors could be linked with declines in catch in the early twentieth century (figure 3). First, global markets for cod were dominated by the more highly regarded Atlantic cod, with Pacific cod described as an inferior product that would spoil quickly [42]. Fisheries for Pacific cod were only competitive when there were shortages in Atlantic cod, particularly during World War I (WWI) when Atlantic fishing operations were interrupted. The domestic market was limited to ‘west of the Rocky Mountains’, with failed attempts to establish permanent markets in the American midwest and Canadian prairies [43]. American vessels also competed with Japanese-caught fish, with a particularly strong influx of Japanese cod in 1919 [44], the same year that demand slumped following WWI.

Market prices for Pacific cod were low, with a ‘very measurable constriction of demand’ [45] when prices rose. Relative to Japanese competitors, wages in the American schooner industry were higher, reducing the overall profitability of the industry and the ability to compete with Japanese products. In 1921, it cost twice as much to produce American salt cod as Japanese salt cod, attributed to the monthly wage of an American fisherman being equal to a Japanese fisherman’s wage for the entire season [46]. Wage conflicts between company owners and the fisherman’s union interrupted fishing, with several American companies halting fishing in 1921 [47]. Shifting consumer preference away from salt cod and towards canned, fresh and frozen fish also reduced overall demand [48], with observations in 1927 that the salt cod era was coming to an end [49].

Finally, changes to fishing technology made it difficult for the Pacific cod industry to adapt. The Japanese fishery began to transition from a schooner-based fleet to trawling vessels in the late 1920s, with the first observation of Japanese trawling in the BS in 1929 and increasing presence throughout the 1930s [50]. Development in the American trawling fleet was slowed by the rugged seafloor in the AI [51] and restrictions on trawling in Alaska waters until 1942. Taken together, the industry’s inability to adapt to these diverse social and economic factors resulted in a reduction in fishing efforts across this time period. By the early 1930s, the San Francisco and Puget Sound fleet had declined by more than half—to fewer than 10 vessels—and by 1938, the San Francisco-based companies had ceased operations permanently.

### (d) Observations of depletion of cod populations

While social factors contributed to catch declines, we also found observations of declining abundance and shifting distribution (table 1). Evidence from shore stations indicated localized declines in abundance in the 1910s; the *Pacific Fisherman* describes previously abundant inshore populations as depleted, while fish remained abundant on offshore banks. Observations in the mid-1920s describe the slow but substantial decrease in the abundance of cod in the previously productive fishing areas in the Shumagin and Sanak Islands, key locations for fishing (figure 1A). In the late 1920s and 1930s, observations describe an inability to locate concentrated cod populations, with decreased abundance of cod noted in the BS in 1927, difficulty in finding concentrated cod in 1936, and decreased localized abundances in particular locations throughout the time period. Observations included speculation that cod may be moving in response to the discard of fish remains overboard and that the activities of the relatively new Japanese trawling fleet may have scattered the schools.

**Table 1.** Qualitative evidence of depletion as measured by observations of reduced abundance and changing distribution.

year	observation
1915	‘...larger vessels usually anchor on the banks and send out their dories, moving the vessel only when the fish show signs of exhaustion in the spot being fished’ [52, p. 21]
1919	‘eventually the inshore banks began to show signs of depletion and in order to keep up the catch it was found necessary to go farther and farther away from the shore stations. ... parts of the banks which had not been fished before, and where fish was still abundant... On the inshore banks fishing was restricted to sheltered areas within about five miles of the stations, as fish were abundant here…’ [53, p. 62]
1925	‘shore stations in the Shumagin Islands and on the island of Sanak… were very profitable for many years, but then the supply of fish diminished drastically… over many seasons ... Similar conditions occurred on Slime Bank [Unimak Pass], although not as drastic’ [38, p. 193]
1928	‘.. shortage of fish in the Bering Sea, resulted in a marked decrease in the Pacific codfish production in 1927’ [54, p. 192]
1930s	‘..a schooner seldom remained on the same location for more than three days. It appeared that the offal thrown overboard during several days of fishing caused the fish to move elsewhere’ [38, p. 119]
1935	‘the *Azalea* found fish a little more scattered than in 1933…’ [55, p. 60]
1935	‘... the dory fishermen [in the Shumagin and Sanak Islands] no longer find codfish abundant on the banks [the Shumagin and Sanak Islands] within reach of their small craft’ [56, p. 52]
1936	‘..good fortune in finding spots of codfish was lacking and the whole was most disappointing’ [57, p. 66]
1936	‘the fish seemed to be badly scattered and the boats had extreme difficulty in getting onto profitable spots. Some of the fishermen are of the belief that Japanese trawlers have scattered the schools, making it difficult to make profitable catches from dories’ [51, p. 225]
1937	‘the last year we sent vessels to the Bering Sea, there were few if any cod south of [Amak] Island. For reasons unknown to us, the abundance of fish in different locations would change’ [38, p. 136-137]
1930s	‘Slime Bank (Unimak Pass) produced good yields before the 1940s’ [38, p. 136]

Perhaps most notably, observations of declining size also suggest depletion (table 2). Consistent and quantitative records of average size were kept by fishing companies, as annual fish catches were reported in both numbers and total weight. These records suggest an acute period of declining fish size between 1922 and 1931, with reported declines in the average size of individual fish of up to 25% [68]. In 1923, four news stories reported decline, one of which noted that ‘the fish taken by a number of vessels averaged fully a pound less in weight than the year before’ [60, p. 108]. This trend reversed in 1932 with one article reporting ‘…it appears the fish averaged perhaps a quarter-pound more than the year before… This was somewhat reassuring after a downward tendency of average weights for several years’ [65, p. 181]. Reports of fish maintaining these larger sizes continued through the end of the 1930s.

**Table 2.** Qualitative evidence of depletion as measured by observations of reduced body size. (The number of articles in each year noting a size increase or decrease, with an example of that observation in each year.)

year	size increase	size decrease	example observation
1921	1	0	‘fishing was exceptionally good, and the vessels got much better fare than usual, the fish being large in size as well as numerous’ [58, p. 193]
1922	0	2	‘the total catch … was .. more than in 1921, but the fish were somewhat smaller on the average’ [59, p. 99]
1923	0	4	‘[t]he fish taken by a number of vessels averaged fully a pound less in weight than the year before’ [60, p. 108]
1924	1	1	‘reports early in the season indicated that the codfish taken in Bering Sea by the Puget Sound fleet were of large size, but on weighing out the results have proven very disappointing’ [61, p. 46]
1925	0	1	‘… the fish have averaged smaller in size than usual’ [62, p. 46]
1926	0	1	‘…the majority of the fish ran to rather small sizes this year’ [63, p. 28]
1931	0	1	‘.. there appears to be a gradual decline in the average weight of the Bering Sea codfish when noted over a period of years’ [64, p. 277-8]
1932	1	0	‘…it appears the fish averaged perhaps a quarter-pound more than the year before… This was somewhat reassuring after a downward tendency of average weights for several years’ [65, p. 181]
1934	2	1	‘this apparent paradox, wherein fewer fish gave a greater tonnage is traceable to the average weight of the fish’ [66, p. 199]
1939	3	0	‘the number of fish, however, showed a material decline, which traced to the fact that the individual fish were much larger’ [67, p. 301]

### (e) Overfishing as a driver of decline

**Figure 4.**
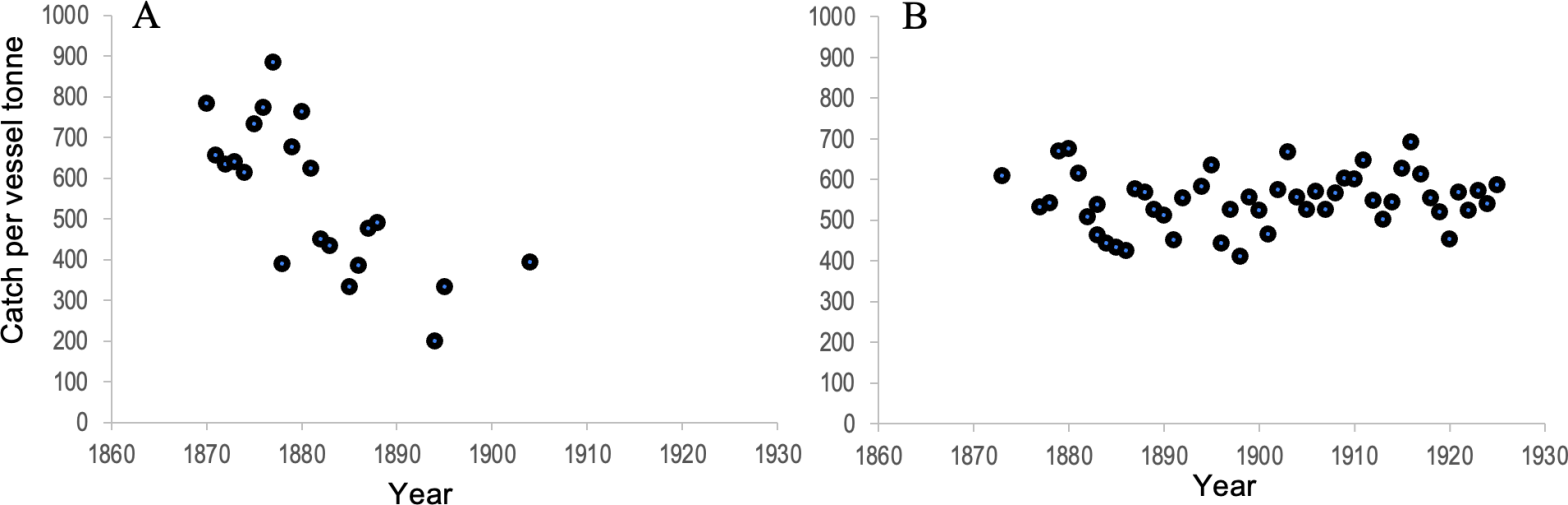
Catch per vessel tonne. Annual averages for (A) the Shumagin Islands and (B) all other fishing grounds, 1870−1925. After 1915, Shumagin Island data are combined with all other data in the original dataset.

Qualitative observations of declining abundance are supported by quantitative evidence for localized, inshore declines in CPUE. Declining trends were observed in the Shumagin Islands, where the average number of fish per vessel tonne declined by more than half, from an average of 784 in 1870 to 393 in 1904 (figure 4A). By contrast, trends in the other offshore regions were stable across this time period, with an average of 549 fish per vessel tonne between 1870 and 1925 (figure 4B). The difference in catch rate supports contemporary qualitative observations that the Shumagin Islands were highly productive at the start of the fishery, but that localized inshore depletions occurred, which were not reflected at the regional level.

Additionally, the relatively low magnitude of historical catch as compared to modern fisheries makes overfishing unlikely as a primary driver of the decline in cod catches observed in the early twentieth century. At its peak in 1919, we estimated that the salt cod fishery caught 20 293 t of cod. By contrast, the average catch in the last decade of available data (2013−2022) was more than an order of magnitude greater at 251 729 t [32].

### (f) Climate drivers of decline

**Figure 5.**
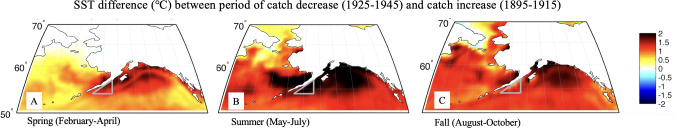
Differences in SST between periods of catch decrease (1925−1945) and catch increase (1895−1915) in the cod fishery show warming in the later period, particularly in summer months. Average temperature differences are shown for (A) spring (February–April), (B) summer (May–July) and (C) autumn (August–October). The white triangle is the reference region represented in figure 6. Differences in water column temperature across the same years and seasons are shown in the electronic supplementary material, figure S4.

We found spatial trends which suggest that the period of catch decline was characterized by warm waters across key seasons and depths relative to cod life history. We considered both surface temperature and water column temperature (0−300 m) across three seasons: spring (February–April), summer (May–July) and autumn (August–October). Warming was strongest for SSTs in the summer and autumn, with temperatures up to 2.0℃ higher in 1925−1945 as compared to 1895−1915 (figure 5). The warming is particularly prominent in the GOA as well as the BS.

**Figure 6.**
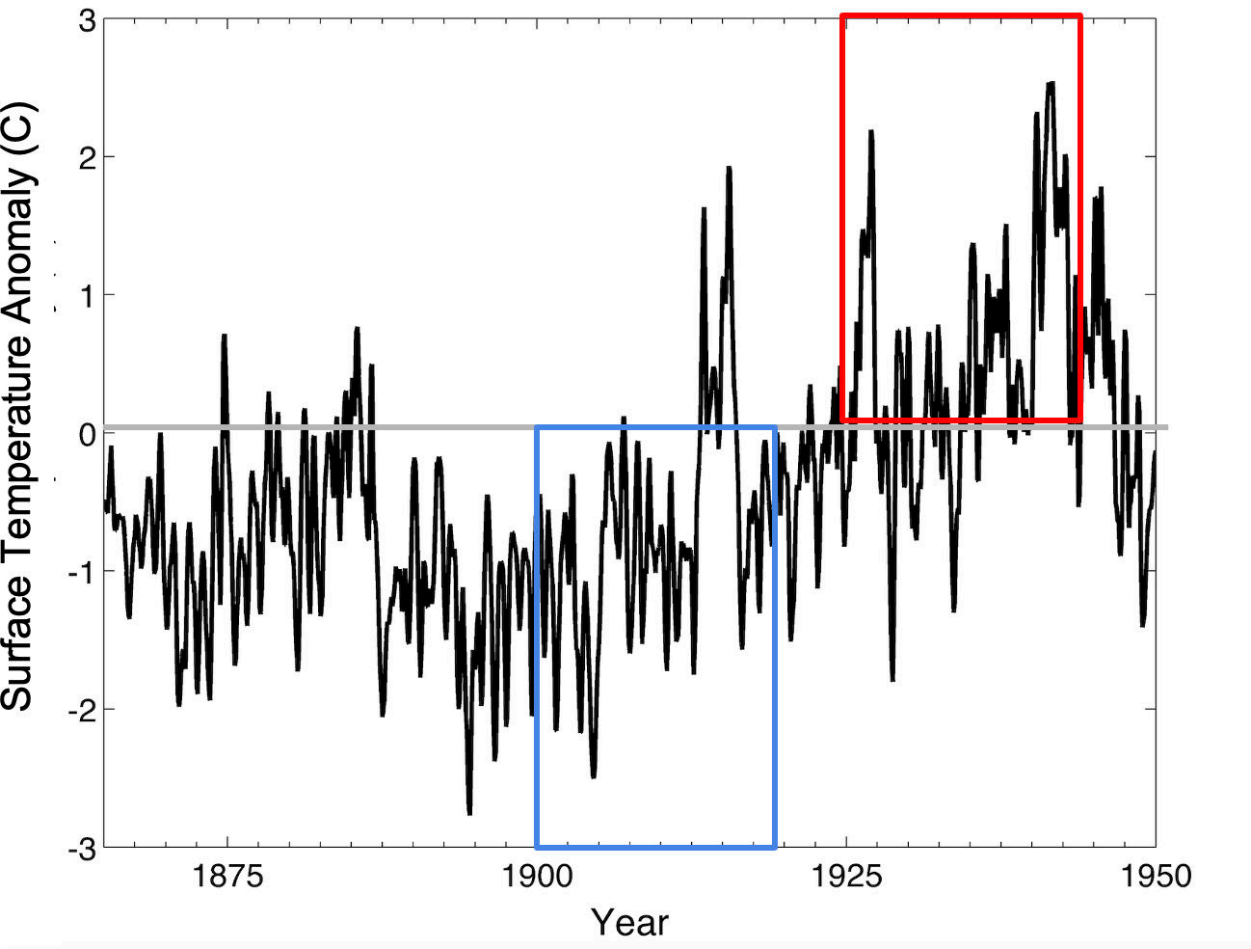
Temporal trends in temperature in the GOA (reference region indicated by the white triangle in figure 5). Sea surface temperature (SST) anomaly for the years of our catch reconstruction (1864−1950). The blue box corresponds to the period of catch increase (1895−1915), and the red box corresponds to the period of catch decrease (1925−1945) used in spatial analysis (figure 5), with a 5-year time lag to account for the time it would take for affected year classes to mature into the fishery. Full temperature time series are shown in the electronic supplementary material, figure S5, and SST in °C are shown in the electronic supplementary material, figure S6.

We also found a temporal signal of warming along the coastal shelf south of the Alaska Peninsula that corresponds to the period of decline in the cod fishery. Surface temperatures in the period before 1920 were characterized by anomalously cool waters, while those in the period of decline (1925−1945) were characterized by anomalously warm surface temperatures (figure 6). These patterns were similar across seasons but with more variability in the autumn. With regard to water column temperature, we found much weaker signals with maximum anomalies confined to the near-shelf region south of the Alaska Peninsula during spring (electronic supplementary material, figure S4). In absolute terms, SST peaked at 12°C (electronic supplementary material, figure S6), which are above temperature thresholds known to affect metabolic rate [69].

To understand whether the temperature variations identified here are influenced by multi-decadal periodicity associated with the Pacific Decadal Oscillation (PDO) and/or North Pacific Gyre Oscillation (NPGO) atmosphere-ocean dynamics [70], we compared the GOA SST time-series with time-series of the PDO and NPGO (electronic supplementary material, figures S7 and S8). We find that there is a correlation between the GOA SSTs with the PDO, although it is relatively low and does not explain much of the variance (*r* = 0.37, variance explained is 14%; electronic supplementary material, table S4). Furthermore, while there appears to be good correspondence between the two during the 1925−1945 period (with both indicating warm conditions), the same correspondence does not hold during the 1895−1915 period, during which the PDO indicated warm conditions but the Alaska Peninsula SSTs were at their coldest, indicating that local factors other than decadal fluctuations in the basin-scale temperature patterns captured by the PDO and/or NPGO affected temperatures.

## 4. Discussion

Unprecedented levels of increasing atmospheric carbon dioxide have directly affected the world’s oceans through increasing temperatures, changing current patterns and ocean acidification, often more quickly than we initially estimated [71]. Fishery managers are grappling with unprecedented change that often occurs as anomalies, without warning [10]. Here, we find historical precedent for declines in the Pacific cod fishery from the recent historical past that may help explain current events and predict future trends.

Our analysis of diverse drivers of change suggests a combination of social and environmental factors that affected the decline in cod catch in the 1920s and 1930s. Social changes certainly affected the fishery decline, with the declining market for salt cod being a clear driver of catch declines. Yet we also found evidence for localized overfishing, particularly in regions that had been targeted consistently for decades, like the Shumagin Islands, with evidence for declining relative abundance. We also found strong evidence for declines in individual body size, which can be a consequence of overfishing [72]. Changes in body size and distribution have been documented in comparisons of archaeological and modern data from this same region, with areas that have been consistently targeted demonstrating declines [73]. Intensive fishing over 50 years in the Shumagin Islands and inshore areas could have resulted in localized depletions. Finally, our analysis of water temperatures suggests that the impacts of warming could have helped to push a fishery that was already unstable because of localized overfishing and economic marginalization to collapse. Along with our finding that increased temperatures coincided with reduced catch, our time-series analysis suggests that natural forcing played a role in the observed fluctuations and that there were impacts on the population beyond simple human fishing pressure. Social and ecological stressors were probably intertwined; for example, decreasing body size could have resulted in inefficiencies in fish processing, leading to declines in profitability. Gaps in the historical data preclude more highly refined analyses of factors such as shifting distribution and variable recruitment that may have also contributed to changes in the early twentieth-century fishery.

In many ways, the collapse of the Pacific cod fishery in the 1930s provides an ocean analogue to the Dust Bowl. This well-known phenomenon in environmental history was a complex social and ecological event, associated with warming in the 1930s [74,75]. This same warming existed in the ocean, with the 1930s and 1940s as a period of warming around the world [10]. Yet climate histories of marine fisheries from this period have not been well studied. This case study suggests that there is an opportunity to investigate the linked social and ecological drivers of change to fisheries in the early twentieth century, which was both a period of dynamic social change and ocean warming.

This analysis of multiple drivers draws from both qualitative and quantitative evidence, underscoring the need to consider multiple types of information in evaluating complex social–ecological systems and emphasizing the need for additional perspectives. For example, biological data stored in historical fish samples could further illuminate how cod populations responded to warming conditions in the twentieth century. Museum collections curated in the nineteenth and early twentieth centuries contain cod skeletons with preserved DNA and chemical signatures, such as palaeoecological data that are available in the isotopic (*δ*^13^C, *δ*^15^N, *δ*^18^O) and trace element (Ba : Ca, Sr : Ca) composition of extracted bone collagen and otolith calcium carbonate (CaCO_3_). Otoliths are accretionary structures whose chemistry reflects fish movement and migration, water temperature and salinity [76], whereas the chemistry of individual amino acids within preserved fish bone collagen records local primary productivity and fish habitat use [77]. These sources could tell us how cod populations responded to changing temperature conditions and could be used for testing the localized effects of changing temperature on cod feeding activity. Ancient DNA analysis complements this record by generating snapshots of genetic diversity and population structure at specific points in time, which can be used to examine shifts in population distributions or breeding locations in response to past environmental changes.

Our historical archival work also links to traditional ecological knowledge of contemporary Aleut (Unanga$\hat{x}$) fishermen who still live in and fish in this region and whose Indigenous language gives clues into social–ecological systems over time. The name for cod, ‘the fish that stop’ indicates a longer record of variability, while the Shumagins nicknamed the ‘Cod Islands’ by the settler cod fishing fleet underscores that communities and cultural identities are substantially dependent on healthy fisheries [78]. Modern fishing communities are only one generation removed from the ‘dory days’ of the early twentieth century, as their Scandinavian and Indigenous fathers and grandfathers fished the schooner-dory fisheries and worked at the cod stations. Their Indigenous mothers and grandmothers processed and salted cod for markets [79]. Oral histories document cod declines in the 1920s and 1930s, with interviews consistently describing cod as not there, no matter what gear and capacity the fishermen had. Fishing communities also remember their eventual return in the 1960s, with fishermen so excited to see them again that work would usually stop so they could cook and enjoy the cod. This recent history serves as a warning for an uncertain warming future. Even though Pacific cod have returned to their waters for now, communities are still in recovery from the recent cod declines and closure.

Finally, this work supports the broader idea that productivity has been subjected to phase shifts over the past century and highlights the need for additional historical analyses that link fisheries and climate history. For example, for Atlantic cod, periods of higher and lower productivity are known and linked with climatic conditions, with warmer periods tending to positively affect productivity for northernmost stocks [80], whereas the reverse was the case in southern stocks [81,82]. Atlantic cod (in the North Sea) has also shifted its distribution in response to both climate change and fishing pressure [83]. Particularly relevant to modern fisheries management are temperature fluctuations in the twentieth century, with the 1940s representing a period of warming, followed by cooling in the 1960s and 1970s around the world [10]. Notably, many modern fisheries management institutions, including in the United States, began during the 1970s, a period of relative cooling that probably corresponded to higher productivity relative to earlier decades. As temperatures warm beyond those experienced by many modern fisheries, there is a need to look to the past for a broader range of variability experienced by fisheries to help calibrate modern expectations. In this context, it is increasingly important to understand how marine species, ecosystems and coastal communities adapted to past analogues of rapid temperature changes.

## Data Availability

All relevant data are included in the electronic supplemental material [84].
